# Evidence for Choosing Qigong as an Integrated Intervention in Cancer Care: An Umbrella Review

**DOI:** 10.3390/cancers15041176

**Published:** 2023-02-12

**Authors:** Ketti Mazzocco, Alessandra Milani, Chiara Ciccarelli, Chiara Marzorati, Gabriella Pravettoni

**Affiliations:** 1 Department of Oncology and Hemato-Oncology, University of Milan, Via Santa Sofia 9/1, 20122 Milan, Italy; 2 Applied Research Division for Cognitive and Psychological Science, European Institute of Oncology, IEO IRCCS, Via Giuseppe Ripamonti 435, 20141 Milan, Italy; 3 Nursing School, European Institute of Oncology, IEO IRCCS, Via Giuseppe Ripamonti 435, 20141 Milan, Italy

**Keywords:** Qigong, mind–body, integrated care, integrative medicine, integrative nursing, cancer care management, psychology, cancer-related symptom, biomarker, umbrella review

## Abstract

**Simple Summary:**

Cancer is the second leading cause of noncommunicable disease death worldwide. Qigong practice can moderate non-intrinsic risk factors that act on the stress response. The purpose of this umbrella review is to provide a concise summary to facilitate an evidence-based decision on integrating Qigong into cancer patients’ care. Qigong can be considered a safe and suitable mind–body intervention that could be integrated into cancer care management. For cancer-related fatigue, overall quality of life, and cognitive impairment, Qigong showed convincing evidence of a significant effect. However, the results of this umbrella review should be interpreted with caution due to the included studies’ limitations. Higher-quality clinical trials in cancer patients considering Qigong’s direct and indirect outcomes and biological markers are needed.

**Abstract:**

Cancer is the second leading cause of noncommunicable disease death, with an increasing incidence. Qigong practice can moderate non-intrinsic, modifiable risk factors that act on the stress response using physical movements, breathing, and focused attention. The purpose of this umbrella review is to provide a concise summary to facilitate an evidence-based decision to integrate Qigong into cancer patients’ care. Relevant systematic reviews and meta-analyses were identified and retrieved from the JBI database, Cochrane Library, PubMed, Embase, and CINAHL. Of all of the studies assessed, none found evidence of a risk to cancer patients, indicating that Qigong is a safe practice that can be used even by frail patients. The overall quality of life, cancer-related fatigue, and cognitive impairment were improved by Qigong. Different Qigong programs have different impacts on sleep quality and gastrointestinal problems, suggesting that longer practice sessions are required to achieve improvements. To maintain Qigong’s effectiveness, an ordinary practice is essential, or such effectiveness will wear off. The use of biological markers in efficacy assessments needs to be more systematically studied. However, positive WBC, RBC, and CRP trends in Qigong practitioners are evident. Higher-quality clinical studies are necessary to measure variables more closely related to Qigong functioning and consider cancer’s multifactorial nature.

## 1. Introduction

Cancer is the second leading cause of death among noncommunicable diseases, preceded only by cardiovascular diseases [1]. In recent decades, there has been an increase in cancer incidence, and a 60% further increase is estimated to take place by 2040 [1,2]. Unfortunately, such an increase also occurred in younger age groups, among adolescents and young adults, with a 30% increase from 1973 to 2015 [3]. According to an American Cancer Society report, for example, people born in 1990 have twice the risk of developing colon cancer and four times the risk of developing rectal cancer than people born in 1950, with a 2% increase in risk each year in individuals younger than 50 years of age. These data are especially critical when we consider that, while cancers with the highest incidence remain breast, prostate, lung, and colorectal, the latter two, along with liver, pancreas, and stomach, account for the highest number of cancer-related deaths [2]. Despite progress in early detection, treatment, and progression, certain cancer types continue to increase in incidence in different parts of the world, probably due to longer lifespans and changing patterns of cancer risk factors [4]. Wu and colleagues effectively described the multifactorial nature of cancer, distinguishing between intrinsic, non-modifiable (unavoidable, spontaneous mutations) and non-intrinsic, modifiable risk factors. Among the latter, a further distinction can be made between exogenous factors (e.g., carcinogens, viruses, xenobiotics) and lifestyle factors and endogenous risk factors (e.g., immune system, metabolism, DNA damage response, hormone levels). Exogenous and lifestyle factors are modifiable by acting on the relationship between the individual and the environment or introducing changes in their lifestyles (e.g., physical activity, diet, smoking behavior, and healthy habits), whereas endogenous risk factors are only partially modifiable. Despite this formal distinction among risk factors, a practical distinction is considerably more difficult to support, as intrinsic and non-intrinsic factors are not completely independent of each other, with some non-intrinsic factors affecting gene mutations [4]. 

Cole’s [5] Conserved Transcriptional Response to Adversity model effectively integrates non-intrinsic (exogenous and endogenous) risk factors with intrinsic risk factors. According to his model, an individual’s exposure to adverse environmental, social, and psychological conditions for prolonged periods of time leads to the increased expression of genes involved in inflammation and the decreased expression of genes implicated in antiviral responses. Coherently, adverse social conditions (perceived or actual), such as isolation, social exclusion, perceived loneliness, and feelings of inadequacy or shame, activate the Conserved Transcriptional Response to Adversity that, if unresolved, triggers a silent and stable inflammatory response over time, leading to the imbalance of the immune system to the detriment of the Th1 circuit, generally activated in the fight against cancer cells). Other circuits (Th2 and Th17), generally activated to protect the organism from outside threats, such as parasites and bacteria, are also subjected to the consequences of such imbalance and detriment [6]. Persistent psychosocial stress is an example of a risk factor with adverse health-related effects that largely depend on how individuals envision themselves and their relationships with the world. Psychosocial stressors begin as conceptual—not physical—threats but trigger actual, non-fantastic, biological responses. Such stressors depend on our ability to conceptualize them as threatening, neutral, or positive events that we can easily face. The conceptualization of the stressor will help the individual define and activate the most adequate (healthy vs. unhealthy) behavioral and emotional responses.

Therefore, considering that cancer is a multifactorial disease, cancer treatment management should necessarily be multidimensional, integrating medical treatment with complementary interventions able to act on non-intrinsic factors. In addition to traditionally suggested interventions, such as psychological support or psychotherapy, physical activity, diet, and healthy habits (e.g., smoking cessation), mind–body interventions are gaining increasing interest in cancer management, with a particular focus on managing stress associated with the cancer diagnosis, oncology treatments, and related short- and long-term side effects. A systematic review on interventions that affect the interaction between psychological, neurological, endocrine, and immune systems highlighted the significant effect of mind–body activities (such as yoga, meditation, Tai Chi, and mindfulness) on stress-related hormones, inflammatory processes, pro-inflammatory cytokines, and mood and sleep disorders in various diseases, including cancer [7]. 

However, the literature is still far from providing consistent, robust, and high-quality results on the efficacy of mind–body interventions in side-effect mitigation, quality-of-life improvement, and risk-of-recurrence reduction in cancer patients.

The present umbrella review aims to bring order to the existing findings on mind–body interventions for cancer management and to provide a clear overview to support integrated, evidence-based decision making. Considering the large variability in mind–body interventions, we decided that the current review would focus on the Qigong practice. This decision was made based on the hypothesis that Qigong can moderate non-intrinsic risk factors acting on the individual’s stress response through gentle physical movements, breathing, and focused attention, which make the individual’s self-awareness increase, with positive effects on their body tension, breathing habits, thoughts, and emotions. Further supporting our decision is the ease of the Qigong practice, which can be comfortably performed by frail patients, even sitting or lying down. 

### Qigong

Qigong (pronounced “chee gung”) is one of the essential elements of traditional Chinese culture. Qigong therapy, an important branch of Traditional Chinese Medicine (TCM), has a thousand-year history. It is still used today to prevent diseases and treat illnesses, drawing increasing attention from scientists and practitioners of modern medicine. From the early 1990s onward, theories and methods of Qigong have been analyzed and organized, and Qigong has developed as a stand-alone discipline [8].

“Qi” stands for the life energy that flows inside the human body; “Gong” stands for regular work. Hence, Qigong refers to the regular work on internal life energy. 

Considering the diversity of its schools, theories, and methods, “Qigong” is not easy to define. There are different perspectives about its connotations, and hundreds of schools of thought still argue over its proper meaning. The different schools and styles of Qigong all fit into one of three main categories: (1) Martial Qigong; (2) Spiritual Qigong, and (3) Medical Qigong. While Martial Qigong focuses on physical prowess, Spiritual Qigong, inspired by Buddhism, Taoism, and Confucianism, pursues enlightenment through self-awareness, tranquility, and harmony with nature and the Self. Finally, Medical Qigong aims at promoting health and preventing and managing diseases. A consistent definition of Medical Qigong is gradually emerging in the academic literature on Chinese Medical Qigong. A definition proposed by Liu in his book on Medical Chinese Qigong [8] incorporates key ideas from classical Qigong with modern scientific knowledge criteria: “Qigong is the skill of body-mind exercise that integrates the three adjustments of body, breath, and mind into one”, necessary to reach a state of harmonious unity. More specifically, the three adjustments are processes through which the person (1) regulates their posture (releases body tension), (2) harmonizes their breath, and (3) calms their mind. By applying Qigong in the context of modern science, Liu defines it as a mind–body medicine, where training is both physical and mental. To effectively unify the body, mind, and breath into one, the individual needs to master practice skills and techniques. Medical Qigong incorporates a multitude of Qigong styles, the most remarkable ones usually being the Five-Animal Frolics, the Six-Syllable Formula, and the Baduanjin (Eight Pieces of Brocade) [8].

In contrast to other physical exercises that focus on the outer body, operating on stretching, reinforcing, contracting, and relaxing the muscles and joints, Qigong emphasizes the importance of the inner body, developing and improving enteroception and self-awareness of the whole body, which in turn guide the Qigong practitioner to reach and maintain mind–body relaxation [8]. Furthermore, compared to other mind–body activities, Qigong is suitable for frail people or people not inclined to endurance or vigorous activities [9].

Many studies have been performed investigating Qigong’s beneficial effects on cancer patients’ well-being. There are also numerous systematic reviews and meta-analyses on specific cancer types, treatment-related symptoms, or different outcomes, from quality of life to sleep disturbance. There are no concise assessments that integrate the increasing evidence to guide decision makers in the integrated care of cancer patients. Only two other overviews [10,11], to our knowledge, have been conducted in this area, but none encompassed all oncological conditions and all possible outcomes.

The present umbrella review aims to synthesize results from different reviews and meta-analyses and to provide a more consolidated overview on the effect of Qigong as an integrated intervention for cancer management. The results of the current umbrella review will be organized to support decisions about whether, when, and how Qigong can be considered and prescribed as an integrated intervention to optimize cancer patients’ care management.

## 2. Materials and Methods

### 2.1. Umbrella Review Methods

An umbrella review is a synthesis of existing systematic reviews, and it is designed to incorporate all types of syntheses of research evidence, including systematic reviews in their various forms (e.g., effectiveness, meta-aggregative, integrative) and meta-analyses to provide a comprehensive examination of a body of information available on a given topic. The current umbrella review was conducted following the JBI Manual for Evidence Synthesis [12] and according to the Preferred Reporting Items for Systematic Reviews and Meta-Analyses (PRISMA) guidelines. No institutional review board approval was necessary.

### 2.2. Inclusion Criteria

#### 2.2.1. Participants

This umbrella review considered reviews reporting on cancer patients. No restriction was applied on participants’ age, sex, type of cancer, stage, or treatment.

#### 2.2.2. Phenomena of Interest

The phenomena of interest in the current review are Qigong’s effects on cancer patients. For this study, an “effect” was defined as a significant difference in a specific outcome—objective or subjective—measured by adequate tools, including questionnaires, performance tasks, blood tests, and other measurement methods. We accepted terms similar to Qigong (e.g., Qi Gong, QE as qigong exercise, Baduanjin, Medical Qigong, Six Healing Sounds, Chinese Qigong). We excluded reviews that (i) did not report analyses or results on a specific Qigong intervention; (ii) did not refer to cancer patients; (iii) were not in English.

#### 2.2.3. Types of Studies

We included reviews labeled as systematic reviews and/or meta-analyses that identified and reported the effects of Qigong in cancer patients. We excluded narrative reviews, primary studies, protocols, theses, and conference papers.

### 2.3. Information Sources, Search Strategy, and Data Extraction

A search strategy with Population, Intervention, Comparator, and Outcome (PICO) components was used to identify and retrieve articles. Four databases, including the JBI database, Cochrane Library, PubMed, Embase, and CINAHL (Cumulative Index to Nursing and Allied Health Literature), were systematically searched from inception through December 2022. The search was limited to English. The PICO components included a list of keywords and MeSH terms (MeSH terms for PubMed) such as Qigong OR Qi gong, Neoplasm, Cancer, Systematic Review OR Meta-Analysis. The final search results were entered into Ryyan Software, automatically screened for duplicates, and later screened manually for accuracy. Two reviewers (AM and CC), independently and in a blinded manner, screened titles and abstracts for eligibility, according to the criteria described in Section 2.2.2 (Phenomena of Interest). Then, they independently reviewed the full texts of potentially relevant studies. A third reviewer (KM) arbitrated disagreements that could not be resolved through consensus.

### 2.4. Assessment of the Methodological Quality of Systematic Reviews

Two reviewers (AM and CC) independently evaluated the included systematic reviews using the A Measurement Tool to Assess Systematic Reviews (AMSTAR 2). AMSTAR 2 is a quality assessment and risk-of-bias tool composed of 16 criteria. Each systematic review and meta-analysis was evaluated by verifying its compliance with AMSTAR 2’s criteria.

Discrepancies were resolved through discussion. It was established, a priori, not to exclude any reviews based on quality assessment, but to present all quality assessment results. This is coherent with the current umbrella review’s aim of providing a comprehensive overview of the available evidence on the phenomena of interest to facilitate healthcare professionals’ decision-making processes in clinical practice. Therefore, we considered it crucial that all relevant literature, regardless of its quality, be included and systematized in the present manuscript. Being provided with the quality assessment of all included papers, the reader will possess the necessary information to eventually choose which data are worth taking into account for decision-making purposes.

## 3. Results

### 3.1. Search Strategy Outcome

**Figure 1:**
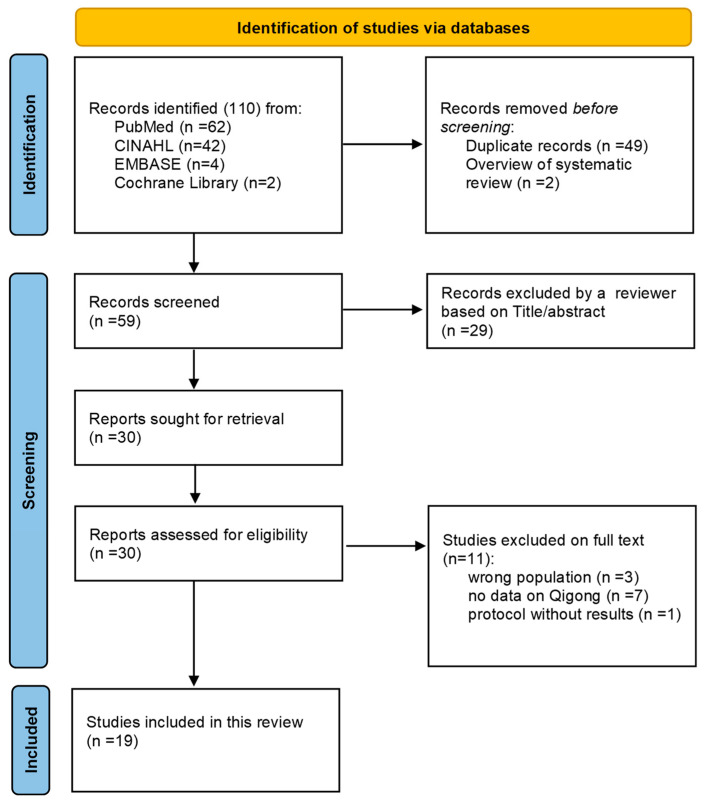
PRISMA flow chart describing the selection of included publications.

Nineteen articles, including seven systematic reviews [13,14,15,16,17,18,19] and twelve meta-analyses [20,21,22,23,24,25,26,27,28,29,30,31], met the established inclusion criteria and were evaluated (Figure 1, Table 1).

### 3.2. Qigong Styles

The studies included in the present work showed high heterogeneity in the Qigong styles used in the original clinical trials (for details on the styles, see Table 1). The most common styles mentioned are Baduanjin [23,24,26,28,30], Chan-Chuan Qigong [17,18,24,26], and Guolin Qigong [13,17,18,24,25,26]. When not better specified, Medical Qigong [13,14,17,18,19,25,31], Qigong exercises [17,18], or Internal Qigong [15,29] were the labels used to indicate the Qigong intervention. Some works did not specify the Qigong style at all but described the Qigong practice as focusing on the synchrony between body posture/movements, breathing regulation, and intentional meditation [16,20,21,22,27]. 

Only a few works reported a comparison among Qigong styles. More specifically, Wang’s meta-analysis [30] showed a trend toward better score improvement in the Baduanjin Qigong group compared to the other Qigong styles. Meng and colleagues [24] showed a greater benefit from Baduanjin compared to the other styles. In contrast, Klein et al.’s systematic review [13] concluded that there was no evidence of the “superiority” of one Qigong style over others. However, this last systematic review did not included studies using Baduanjin as an intervention, and therefore, it cannot be considered to be in opposition to the previously described styles’ comparisons. 

Studies reported in Van Vu et al.’s systematic review [17] were all found to be effective for one or more of the main assessed outcomes; this systematic review concluded that there was no evidence of one Qigong style’s “superiority” over others.

### 3.3. Methodological Quality

Of the 16 quality assessment criteria listed in AMSTAR2 [32], five applicable criteria (31 percent) were met by all 19 included studies. Two meta-analyses [24,27] and two systematic reviews [13,18] met 14 of the 16 criteria, and another four meta-analyses [21,26,28,30] and two systematic reviews [15,19] met 13 of the 16 criteria (see Table 2). The three most violated criteria (Q3 not met in 53%, Q7 not met in 89%, and Q10 not met in 95% of the included studies) do not have a high impact on the research methodological process, but lack the following: (1) a list of excluded studies, (2) the funding of included studies, (3) a statement of the reasons for the study designs included in the reviews. However, criterion Q2, regarding the design and registration of a protocol prior to the review performance, was also violated in 58% of the included studies. This is considered a major violation. However, this violation does not necessarily imply that the authors did not follow common best practice guidelines for the literature review. This considered, the authors concluded that 15 out of 19 secondary studies present moderate–good quality. Only two articles can be considered to be of low quality because they do not meet at least 6 out of 16 criteria [22,31]. In addition, Chan’s paper [18], although meeting 14 of 16 criteria, violates 1 criterion (Q5) that may compromise the research process’s quality (see Table 3).

### 3.4. Outcomes

#### 3.4.1. Quality of Life

Quality of life is defined by the World Health Organization (WHO) as “An individual’s perception of their position in life in the context of the culture in which they live and in relation to their goals, expectations, standards and concerns.” A further definition is provided when considering QoL associated with health and disease (health-related quality of life—HRQOL), where QoL reflects “the impact of disease and treatment on disability and daily functioning” and the “impact of perceived health on the ability to live a fulfilling life” [33].

Among the selected articles, seven meta-analyses and four systematic reviews measured quality of life as an outcome. Six of the meta-analyses considered showed a significant positive effect of Qigong exercise on overall QoL compared to the control group(s) [21,23,24,25,27,28]. No effect of Qigong was reported in the meta-analysis by Tao and colleagues [20], where Qigong was adopted as complementary care in 2 of the 67 included RCTs; one of them measured QoL in a specific population of breast cancer patients after 5 weeks of Qigong practice, while the second one evaluated QoL in a population with different cancer types after 10 weeks of practice. Both studies reported no effects but a high risk of bias.

All the systematic reviews [13,16,17,18] reported at least partial evidence of a significant effect of Qigong practice on QoL. More specifically, one systematic review reported a significant effect of Qigong in 4 of the 5 included studies that used QoL as an outcome [13]; the second one showed a significant effect in 5 of the 8 included studies; a third one reported significant results of Qigong in 6 out of 10 studies [17]; finally, the fourth systematic review [18] showed a higher improvement in the quality of life of Qigong practitioners who presented fewer depressive symptoms at the start of medical treatment [16].

According to Meng and colleagues [24], compared to other forms of Qigong, Baduanjin Qigong might have a greater effect on quality of life, specifically if practiced in a program entailing sessions lasting less than 60 min, with a weekly attendance of at least five sessions, and with a duration of at least 3 months.

Moreover, as indicated in Matthews and colleagues’ meta-analysis [16], among women with fewer depressive symptoms at the onset of XRT, those practicing Qigong reported a better overall QoL compared to those only receiving usual care.

Although the findings reported above were related to the overall quality of life, some studies investigated and reported more specific elements that commonly contribute to determining individuals’ quality of life. In particular, social and family well-being (as measured by the FACT-G questionnaire) presented a stronger positive association with Qigong practice than with control interventions (same study reported in two systematic reviews) [13,18], with Qigong’s positive effect increasing over time. However, a more recent meta-analysis [28] showed no evidence of Qigong practice on social well-being (two studies measuring social functioning with the SF-36 questionnaire and four studies investigating social well-being using the FACT-B questionnaire). Regarding emotional well-being, no evidence of improvement associated with Qigong practice was reported [13,28].

As for functional well-being, measured with the FACT-B, Qigong practitioners significantly improved compared to individuals in the control groups [28].

Evidence on physical well-being and physical functioning is also inconsistent. While Chan et al.’s systematic review [18] included one study reporting Qigong’s significant effect on physical functioning, as measured by the SF-36 questionnaire, Ye’s meta-analysis [28] reported different results depending on the type of questionnaires used. More specifically, of the studies included in the meta-analysis, the four employing the FACT-B reported strong evidence supporting Qigong’s effectiveness, whilst the two studies using the SF-36 showed no significant effect of this practice compared to control interventions.

#### 3.4.2. Sleep Quality

Sleep quality is defined as an individual’s self-satisfaction with all aspects of their sleep experience. Sleep quality includes four aspects: sleep efficiency, sleep latency, sleep duration, and wake-up after sleep onset. Good sleep quality has positive effects, such as feeling rested, having normal reflexes, and having positive relationships. Conversely, poor sleep quality entails consequences such as fatigue, irritability, daytime dysfunction, slowed responses, and increased caffeine/alcohol intake [34].

Sleep quality was reported as an outcome in 8 of the 19 papers included in the present umbrella review. Six of them were meta-analyses [20,21,23,24,26,31] and two were systematic reviews [16,17]. In five articles, Qigong significantly improved sleep quality compared to the control intervention [20,21,23,26,31]. In particular, Cheung’s meta-analysis [26] reported evidence of a Qigong effect on sleep quality after the intervention. The effect appeared to be significantly mediated by a more specific impact of Qigong on fatigue (b = 1.27, SE = 0.24, *p* = 0.002). However, such an effect disappeared 3 months after the end of the intervention.

No evidence of Qigong’s effects on sleep quality was reported in a systematic review and one meta-analysis (respectively, [16,24]). However, conflicting results were reported by Van Vu and colleagues’ systematic review [17], which included three studies with sleep quality as an outcome, one of which claimed Qigong’s effectiveness.

Cheung’s meta-analysis concluded that Qigong’s effects on sleep quality were large but dose-dependent: only more extended Qigong practices (>200 min per week) led to significant effects.

#### 3.4.3. Cancer-Related Fatigue

Cancer-related fatigue can be defined as “a subjective sensation that is disproportional to the widely recognized feeling of being tired […] pervasive and not relieved by rest”, and is characterized by a subjective component often related to objective changes in physical functioning or impaired performance status [35]. In general, fatigue in cancer care is measured as subjective feelings of fatigue, weakness, or lack of energy [36,37].

Nine meta-analyses [21,22,23,24,25,26,28,30,31] and six systematic reviews [12,13,14,15,16,17] reported results on the effect of Qigong on cancer-related fatigue. All studies reported a significant reduction in fatigue in the Qigong’s practitioners compared to controls, except for one meta-analysis [24], where no evidence of such a difference was reported.

Based on a comprehensive analysis, Qigong is the optimal intervention to reduce cancer-related fatigue, similarly to multimodal therapy and cognitive behavioral therapy. Furthermore, as shown by Wu [22], Qigong is far more effective than yoga interventions on such symptoms. In Kuo’s meta-analysis [23], where—in terms of Qigong style—only homogeneous RCTs were included, Baduanjin led to a highly significant reduction in cancer-related fatigue, with no heterogeneity among studies (I-squared = 0%).

The systematic review performed by Matthews [16] provided evidence of a significant fatigue reduction in the Qigong group compared to the control group, both after the intervention and at the 3-month follow-up. However, a more recent meta-analysis [26] showed that such effects on fatigue became non-significant 3 months after the end of the intervention. Still, a further detailed analysis of the studies included in Cheung et al.’s meta-analysis [26] reveals that Qigong’s positive effects remained significant beyond the 3-month time in those studies in which the intervention lasted longer (i.e., 12- and 24-week programs).

Moreover, as indicated in Matthews and colleagues’ meta-analysis [16], among women with fewer depressive symptoms at the onset of XRT, those practicing Qi reported lower levels of fatigue (*p* < 0.01) compared to those receiving usual care/treatment as usual (*p* < 0.05).

#### 3.4.4. Depression

Five meta-analyses [21,24,26,28,31] and four systematic reviews [13,14,16,18] included depression as an outcome. Overall, three [21,24,28] of the five meta-analyses and two [14,16] of the four systematic reviews reported evidence of a significant improvement in depression in cancer patients practicing Qigong. Cheung et al.’s meta-analysis [26] showed no evidence supporting Qigong’s effects on depressive symptoms; however, more accurate reflections and considerations should be made about the studies reporting no evidence. For example, Cheung et al.’s analysis was based on four studies, two of which should be considered with caution: one is a master’s thesis reporting unverifiable data [38], and the second one reports results on the mere effects of Qigong, while further interesting results available in the original article on the interaction between intervention types, Qigong dosage, and baseline depressive symptoms were neither reported nor discussed [39]. In Zeng and colleagues’ meta-analysis [31], three studies were analyzed to assess changes in depression symptoms from baseline to the 12-week follow-up. Two studies significantly favored Qigong over the control interventions, while the remaining study (a master’s thesis) [40] did not report either significant results supporting Qigong’s effectiveness or the control interventions. In a systematic review [13], three out of five included papers reported no evidence favoring Qigong over other interventions; however, it should be highlighted that one of the three studies did not measure Qigong’s but rather Tai Chi’s effects on depression [41]. Larkey and colleagues [42] compared the “sham Qigong” group with the “Tai Chi + Qigong” group (with no actual control group), and reported an improvement in depressive symptoms in both groups. Considering that Qigong exercises were present in both interventions, Klein’s conclusion that there is no evidence suggesting Qigong’s effectiveness on depression should be revised. Therefore, only one [43] out of five studies considered by Klein et al. can be said to have clear and unquestionable evidence against the employment of Qigong practice for patients with depression. It is noted that the one study presenting such evidence [43] adopted a Qigong program entailing 90 min of weekly practice over 8 weeks.

#### 3.4.5. Anxiety

The American Psychological Association defines anxiety as an emotional state “characterized by apprehension and somatic symptoms of tension in which an individual anticipates impending danger, catastrophe, or misfortune” (APA, Dictionary of Psychology) [44]. Anxious individuals generally have recurring intrusive thoughts or concerns and tend to avoid situations due to worry.

Three meta-analyses [24,28,31] and two systematic reviews [13,18] reported results on Qigong’s effects on anxiety. The two systematic reviews and one meta-analysis [31] did not report any evidence of Qigong decreasing anxiety in cancer patients. After a more extensive analysis of these papers, however, it emerged that, of four studies reported in Klein’s review [13], one did not consider Qigong as the intervention group, but rather Tai Chi vs. Spiritual group vs. Usual care, which likely impacts Klein’s review results and their interpretation. Zeng [25] and Chan [18] included in their analysis two studies on the effect of Qigong on anxiety levels, and in both cases, Lam’s unpublished master’s thesis was included [40]. The two most recent meta-analyses reported a significant improvement in anxiety symptoms: Ye’s meta-analysis [28] on anxiety included three studies showing a strong effect and low heterogeneity (10%); Meng’s meta-analysis [24] included five studies reporting a weaker but still significant improvement in anxiety and a higher heterogeneity (89%). However, these last two meta-analyses only focused on breast cancer patients.

#### 3.4.6. Stress/Distress

The World Health Organization defines stress as “any type of change that causes physical, emotional or psychological strain” [45]. The change can be caused by internal or external and real or imagined stimuli or challenges [46]. The word “distress” specifically identifies negative stress with unpleasant feelings, physical reactions, and behaviors and is considered the sixth vital sign in cancer patients [47]. Within the current umbrella review, the terms “stress” and “distress” are used interchangeably.

One meta-analysis [31] and two systematic reviews [13,18] reported distress as an outcome. No significant effect of Qigong practice on such an outcome was reported.

#### 3.4.7. Cognitive Impairment

Cognitive impairment due to cancer treatments has been well documented in non-central nervous system (non-CNS) cancer patients. It is estimated that 13% to 70% of patients receiving chemotherapy have measurable cognitive impairment, sometimes with long-term cognitive difficulties. Cognitive impairment is generally related to the domains of memory, attention, executive function, “processing speed”, visual and verbal memory, and language. However, 13% to 35% of patients present cognitive impairment before their oncological treatment starts [48].

One network meta-analysis [29] and three systematic reviews [13,17,19] reported cognitive impairment after oncological treatment onset as an outcome.

Two of the three systematic reviews claimed a significant effect of Qigong practice on cognitive improvement [13,17]. However, the two reviews considered only one study, which is actually the same in both [49]. The third systematic review [19] concluded that Qigong can largely and significantly prevent cancer patients’ cognitive performance decrease—measured as a self-reported outcome and task performance (e.g., task speed and executive functions).

In Liu’s network meta-analysis [29], the evaluation of cognitive outcomes through subjective assessment did not show significant differences between Qigong and other types of non-pharmacological interventions. A significant positive difference was instead reported for the objective outcomes. In particular, objective outcomes indicated Qigong to be more effective than psychotherapy (SMD = −1.27; 95% CI [−2.41, −0.13]). Similarly, compared to music therapy, Qigong showed significantly stronger effects (SMD = −1.69; 95% CI [−0.10, −3.28]). In ranking the effectiveness of objective assessments, Liu et al. [29] concluded that the top three interventions improving cognitive functions are, in order, Qigong, general exercise, and electroacupuncture.

#### 3.4.8. Biological Outcomes

A biological marker (biomarker) is defined as a “biochemical feature or facet that can be used to measure the progress of disease or the effects of treatment” (Webster’s New World Medical Dictionary). The National Institute of Health Biomarkers Definition Working Group defines a biomarker as “a characteristic that is objectively measured and evaluated as an indicator of normal biologic processes, pathogenic processes, or pharmacologic responses to a therapeutic intervention” [50].

Two meta-analyses [24,25] and three systematic reviews [13,17,18] included studies investigating the effect of Qigong practice using biomarkers as outcomes.

The biomarkers reported by the included studies are IL-2, IFN-γ, IL-6, IL-1β, and TNF-α levels, CRP, phagocytic rate, index of immunity, and ANAE (α-Naphthyl butyrate esterase), IgA, IgM, Ing, LAI, EA, C3, C50, CIC, UDS, WBC, RBC, Hb, Cu-Zn SOD, LPO, AFP, ALP, ALT, AST, albumin, globulin, total protein, and cortisol levels.

In Zeng’s meta-analysis [25], four studies presented Qigong as an intervention. Two of them reported a significant reduction in CRP levels in the Qigong group following a 10-week program [49,51], while a third study, where the Qigong program lasted 8 weeks, showed no significant reduction in practitioners’ CRP levels [52]. The fourth study measured the level of cortisol, but no significant differences were found between the Qigong and control groups [39].

The results of Meng’s meta-analysis [24] showed a significant reduction in IL-2, IFN-γ, IL-6, IL-1β, and TNF-α levels in breast cancer patients participating in the Qigong practice group [53,54]. Regarding Lan’s findings [54], they were part of a master’s thesis that is not possible to retrieve. Liu’s paper was retracted in 2020 because of an approval withdrawal by their Institutional Review Board. Despite the retraction, the meta-analysis by Liu et al. [53] showed evidence of Qigong’s effectiveness over the control intervention only with regard to TNF-α levels.

Chan’s systematic review [18] reported strong evidence favoring Qigong practice improving the condition of cancer patients’ biomarkers. The most solid evidence has been demonstrated for white and red blood cells (WBC, RBC) and hemoglobin (Hb): four original studies measuring them found a significant increase in such values in the Qigong group compared to the control group. Evidence was also reported for ANAE, with an increase in values in cancer patients practicing Qigong demonstrated by three independent studies.

In Klein’s systematic review [13], among the seven studies considering Qigong as an intervention, four studies measured biomarkers. Of these, two reported no differences in the cortisol level [39,40] and biomarkers associated with hepatic function [40] between the Qigong and control groups, while in the remaining two studies (the same described in Zeng’s meta-analysis [25]), patients in the Qigong group, compared to those in the control group, presented a significant reduction in CRP levels (*p* < 0.05) [49,51].

#### 3.4.9. Other Cancer-Related Symptoms

This section summarizes those findings that, although reported in the meta-analyses and systematic reviews, were not systematically studied.

Six articles reported results for different cancer-related symptoms. Pain was reported in one meta-analysis [21] and one systematic review [17], with no differences between the Qigong and control groups. Dyspnea was reported to be unaffected by Qigong practice [14,17].

Studies on gastrointestinal symptoms showed evidence of the Qigong practice’s effectiveness in improving appetite and intestinal function [18], nausea, vomiting, and stomatitis [15]. Regarding nausea and vomiting symptoms, two other systematic reviews highlighted conflicting results, depending on the Qigong program’s number of sessions and duration: nausea and vomiting significantly decreased after 4 weeks of practice, while no significant difference between the Qigong and control groups was observed in 8-week programs [17,18].

Strength was mentioned in two systematic reviews [17,18], which reported a significant increase in strength in the Qigong group. Both systematic reviews reported results for only one case–control study, one investigating muscular strength and the other discussing constitutional strength. These results should be interpreted with caution, since they might derive from different types of strength measurements and are both based on a case–control trial design, and one of them is not published in a peer-reviewed journal but as a proceeding of an international conference.

Neuropathy was studied in only one randomized controlled trial reported by Van Vu et al.’s systematic review [17], which highlighted a significant symptom decrease in the Qigong group.

Cardiovascular symptoms (systolic blood pressure) were evaluated in only one randomized clinical trial reported by Klein’s systematic review [13]. The results showed a significant reduction in Qigong practitioners’ systolic blood pressure compared to that of patients in the control group.

The mood state, described as general mood disturbance, was studied in one randomized controlled trial [51] reported in two systematic reviews [13,18]. Mood disturbance was measured by the Profile of Mood State (POMS) questionnaire, which involves six subscales: tension/anxiety, depression, anger and hostility, lack of vigor, fatigue, and confusion. This study showed a significant effect of Qigong practice on general mood disturbance. An analysis of the original trial highlighted substantial differences between the Qigong and control groups with regard to the tension/anxiety, fatigue, and lack of vigor subscales, with the Qigong practitioners presenting a significantly greater improvement.

A synthesis of the evidence in favor or against the effectiveness of the Qigong intervention is provided in Table 4 for psychological and clinical outcomes and in Table 5 for biological outcomes.

## 4. Discussion

This umbrella review evaluated the effects of Qigong practice on cancer patients’ psychological, clinical, and biological outcomes.

No evidence of danger has been reported for cancer patients in any of the analyzed studies, confirming that Qigong is a safe practice that can also be performed by fragile populations such as cancer patients.

Overall, Qigong was found to be more effective than control interventions in improving the overall quality of life and cancer-related fatigue. Its effectiveness on sleep quality and gastrointestinal symptoms (i.e., nausea, vomiting, appetite, and bowel function) appeared to be dependent on the kind of Qigong program. More specifically on this last point, programs involving more than 200 min practice per week and lasting more than 8 weeks have been shown to be the most effective. Furthermore, the results seem to be consistent in suggesting that Qigong’s effectiveness disappears once the individual stops practicing. This is in line with findings from a randomized controlled trial on depression in psychiatric patients, in whom the same mechanism was observed (in intensity and duration): as patients suspended pharmacological antidepressant treatment, its effectiveness disappeared [55].

Another significant effect of Qigong was found for cognitive impairment, mainly when objective measures were used, such as task performance to investigate cognitive speed, memory, attention, and executive functions. Conversely, the effect is controversial when analyzed using self-reported measures. This difference between objective and subjective measures should be further investigated to understand whether patients’ psychological interpretations of their conditions might have affected their perceptions of any effects.

Inconsistent is the evidence about depression and anxiety. As for depression, out of nine studies included in the current umbrella review, four showed no evidence supporting Qigong over other forms of the intervention. A possible explanation for the results’ inconsistency might be the quality of the included studies and the reviews’ and meta-analyses’ primary studies’ data quality and selection. For instance, a better distinction between mind–body disciplines should be considered, specifically between Tai Chi and Qigong, which, in some papers, are treated as interchangeable. Despite these being similar practices, the action mechanism is different: Qigong was born as a health/medical exercise, mainly focusing on an “inside” energy flow, and is easier to master than Tai Chi. On the other hand, Tai Chi was initially a martial art, consisting of movements with fighting functions and, therefore, primarily focusing on “outside” defense and attack intentions while practicing [56].

In addition, some interactive variables that were excluded from the meta-analysis (that is, patients’ depressive symptoms at baseline and the Qigong practice dosage) were demonstrated to influence Qigong’s effectiveness on depression at follow-ups. A similar explanation can also be provided for the results’ inconsistency on the anxiety outcome.

Finally, no evidence was reported on improvement in self-reported stress responses, dyspnea, and pain.

Concerning biological outcomes, robust evidence has been found for WBC, RBC, Hb, and α-Naphthyl butyrate esterase (ANAE), demonstrating that Qigong has a positive effect on the immune system. Clear evidence was also found for Qigong’s effectiveness in reducing inflammation, as indicated by CRP. However, as for other outcomes, the Qigong dosage is paramount, since its effect on CRP is detectable only in Qigong programs lasting at least 10 weeks. No evidence was reported for cortisol levels or for IFN-γ. Because of the type of studies on cytokines, no conclusions can be made on these outcomes. In fact, studies investigating these types of biological outcomes should be considered with caution: one has been retracted [53] and the other was based on a master’s thesis [40].

Given the evidence summarized above, and bearing in mind the need for high-quality studies with larger sample sizes, a definitive, univocal decision is challenging to make on whether, when, and how Qigong can be prescribed to cancer patients. Certainly, Qigong can help patients deal with fatigue and cognitive impairment and improve their quality of life, their sleep quality, and cancer-related symptoms. However, studies conducted so far suggest the use of an allopathic approach to Qigong in cancer patients, consistent with traditional Western medicine, where Qigong is prescribed as a health intervention, independently of other contextual and personal factors, and in contrast to the multifactorial nature of cancer, for which the interaction among systems should be taken into account. Indeed, if we consider cancer to be a multifactorial disease, determining the efficacy of a single intervention without considering all other interacting factors might generate misleading conclusions. In fact, no information about factors such as nutrition, lifestyles and habits, psychological well-being, or social and physical environments was explored in the systematic reviews and meta-analyses included in the present work. The effectiveness (positive, negative, or none) of a mind–body intervention may depend—at least partially—on some of the aforementioned factors. The only additional factor explaining Qigong’s effectiveness was intrinsic to the Qigong intervention itself, that is, the type of program and its dosage.

Another aspect worth discussing is the lack of measurement of cancer patients’ awareness (about tension, breathing habits, thoughts, and emotions) and enteroception, which are the Qigong practice’s most essential outcomes. Enteroception can be defined as the process through which the nervous system (both central and neurovegetative) perceives, interprets, and integrates signals originating from within the body, providing a moment-to-moment mapping of the individual’s internal context at both conscious and unconscious levels [57]. More specifically, this process encompasses the transmission, processing, perception, interpretation, and use of information within the body to prompt appropriate changes to deal with possible detected errors or in response to requests for change and adaptation [58]. From this perspective, the subjective experiential states involved in enteroception are related to the Self and associated with motivation and emotion [59]. As described in the Introduction section, Qigong exercises train the practitioner on all of these aspects through three adjustments, i.e., body, breathing, and mind regulations. Notwithstanding this, awareness and enteroception could be the mediators of Qigong’s effectiveness on other psychological, clinical, and biological outcomes.

## 5. Conclusions

In conclusion, Qigong can be considered a safe and suitable mind–body intervention that could be integrated into the management of cancer care for all patients, regardless of age, sex, and performance status. Some results’ inconsistencies in the present work, however, suggest the importance of looking at cancer patients with the adoption of a wider, bio-psycho-social approach: assessing and taking care of the diverse social, psychological, and biological domains of the cancer patient can, indeed, optimize each intervention and treatment they are prescribed, from the oncological treatment to diet and mind–body practices.

However, the present umbrella review’s results about healthcare professionals’ decision making on Qigong exercise prescription to cancer patients should be interpreted with caution due to the included studies’ limitations, as highlighted in the Discussion. In particular, the choice to omit the first direct outcomes of Qigong practice from the effectiveness evaluation, such as awareness, breathing habits, postural aspects, and enteroception, might have misled systematic reviews’ and meta-analyses’ results and conclusions about Qigong’s effects on multiple indirect outcomes. Therefore, it is paramount to improve the quality of future clinical trials on the matter by planning longer follow-up times, including measures of variables able to reflect cancer’s multifactorial nature and Qigong’s core functioning, and identifying more objective measurements, such as the systematic investigation of biological markers.

## Figures and Tables

**Table 1:** Descriptive summary of secondary studies included in the umbrella review (n 19).

Authors	Study Design	Number of Studies	Population	Qigong Styles in Exposure Group	Control Group	Outcome	Results
Klein 2016 [13]	Systematic review	11 RCTs	831 cancer patients (50% breast)	Guolin Qigong, Kuala Lumpur Qigong, Medical Qigong	Any type of control	Non-biological outcomes (fatigue, QOL, depression, mood, anxiety, distress, sleep quality, cognition, systolic blood pressure, and survival rate). Biological marker outcomes of inflammation and immune function	Significant effect of Qigong on: Fatigue: (*p* < 0.03) in 4/5 studies; Mood: (*p* = 0.021) in 1/1 study; Cognition: (*p* = 0.014) in 1/1 study; Social/family well-being: (*p* = 0.022) in 1/1 study; QoL: (*p* < 0.05) in 4/5 studies. Cardiovascular (systolic BP): (*p* = 0.002); Inflammatory response/immune support: (*p* < 0.05) in 6/10 studies No significant effect of Qigong on: Survival rate, sleep, anxiety, depression (except for 1/5 studies), and distress.
Henshall 2019 [14]	Systematic review	3 RCTs; 7 pre–post test	516 cancer patients (58% lung)	Medical Qigong (style n.r.)	n.r.	Dyspnea, fatigue, depression	Significant effect of Qigong at 10 weeks post-intervention on: Fatigue: (MD = 5.70 [95% CI, 3.32–8.09]) Depression: (MD = 2.56 [95% CI = 5.14–0.01]; *p* = 0.029) No effect of Qigong on: Dyspnea.
Lee 2007 [15]	Systematic review	4 RCTs; 5 CCTs	871 cancer patients	Internal Qigong (style n.r.)	Any type of control	Treatment effects	Significant effect of Qigong on: Fatigue (*p* < 0.05); Nausea, vomiting, and stomatitis (*p* < 0.05); Distress (*p* < 0.01).
Matthews 2018 [16]	Systematic review	12 RCTs; 3 quasi-experimental designs	1691 breast cancer women	Qigong (style n.r.)	Exercise, usual care	Sleep quality	Significant effect of Qigong on: Fatigue: decrease at post-intervention (*p* = 0.005) and 3-month follow-up (*p* = 0.024); decrease in people with fewer depressive symptoms at the RXT onset; Depression: decrease over time in both Qigong + Tai Chi and Qigong Shame (*p* < 0.05) compared to the control group; QoL: better QoL in people with fewer depressive symptoms at the RXT onset. No effect of Qigong on: Sleep quality
Van Vu 2017 [17]	Systematic review	14 RCTs; 7 CCTs; 1 quasi-CCT	1751 cancer patients	Guolin New Qigong, Tai Chi Qigong, 18 forms of Tai Chi Qigong, Yudong Kong exercise, Zhi Neng Qigong, Chan-Chuang Qigong, Medical/exercise Qigong (style n.r.), Sporting Qigong (style n.r.),	Any type of control	Physical symptoms, psychological symptoms, quality of life, adverse events	Significant effect of Qigong on: Fatigue: lower in 7/10 studies; QoL: improved in 6/10 studies; Neuropathy symptoms; Muscular strength; Cervical spine range of motion and shoulder complex range of motion; Frailty; Cognitive function; 6 min walking. No effect of Qigong on: Pain; Dyspnea; Cortisol rhythm; Temporomandibular joint mobility: Tumor size; Survival rate. Conflicting results on: Psychological symptoms; Sleep quality; Gastrointestinal symptoms.
Chan 2012 [18]	Systematic review	8 RCTs; 15 CCTs	Studies on physical and psychosocial outcomes covered 572 cancer patients in Qigong groups and 595 cancer patients in control groups; the studies on biomedical outcomes covered 656 cancer patients in Qigong groups and 601 cancer patients in control groups	Medical/exercise Qigong (style n.r.), Guolin Qigong, Chan-Chuan Qigong, YoudoKong, a series of Qigong exercises (Jing Yang Gong, Fong Song Gong, Zhen Qu Yun Sing Gong, Zi Ti Sun Taiji Gong), multi-style Qigong	Any type of control and treatment	Self-perceived symptoms, quality of life, psychological distress, survival rate, response rate, fatigue, physical functioning, body weight, tumor size, “inflammatory markers” (C-reactive protein), parameters of immunity such as immunoglobulin and complement, the numbers or proportions of blood cells or their antioxidant capacity, and hepatic function	Significant effect of Qigong on: Symptoms (strength, appetite, diarrhea, or irregular defecation); Inflammation; Overall QOL (FACT-G); Mood disturbance; Increase in the number or levels of components in peripheral blood; Increase in the 5-year survival rate. Significant effect of Qigong on: Fatigue; Physical functioning (SF-36); Treatment side effects; Nausea and vomiting after 4 weeks (not significant after 8 weeks). No significant effect on: Psychological distress; Anxiety; Depression; Symptoms after 2.5 months.
Farahani 2022 [19]	Systematic review	9 RCTs; 1 pilot study; 1 feasibility study	1032 cancer survivors (85.8% breast cancer women)	Medical Qigong (style n.r.), Six Healing Sounds	Waitlist control, active control, usual care	Cognitive function	Significant effect of Qigong on: Subjective self-reported cognitive functions (in 6 out of 11 studies): for significant studies *p* < 0.05; Objective cognitive performance (in 1 out of 2 studies): processing speed (*p* = 0.007); executive function (*p* = n.r., Cohen’s d = −0.43 in favor of Qigong group); improvement in cognitive function (*p* = 0.014).
Tao 2016 [20]	Meta-analysis	67 RCTs	5465 adults with cancer	Qigong (style n.r.)	Usual care and active control conditions	HRQOL at post-treatment, cancer-related symptoms, and therapy-related adverse events	Significant effect of Qigong on: Sleep disturbance: positive effect of Qigong on Z = 3.02 (*p* = 0.003). No effect of Qigong on: QoL: Z = 0.98 (*p* = 0.33).
Wayne 2018 [21]	Systematic review and meta-analysis	15 RCTs; 7 studies with non-randomized design or no control group	1571 cancer patients	Qigong (style n.r.)	Active control; no treatment control	Cancer symptoms	Significant effect of Tai Chi Qigong on: Sleep difficulty: reduction in difficulties (Hedges’ g = −0.49, 95% CI −0.89 to −0.09, *p* = 0.018); Fatigue: reduction in fatigue (Hedges’ g = −0.53, 95% CI −0.97 to −0.28, *p* < 0.001); Depression: reduction in depression (Hedges’ g = −0.27, 95% CI −0.44 to −0.11, *p* = 0.001); QoL: (Hedges’ g = 0.33, 95% CI 0.10 to 0.56, *p* = 0.004). No effect of Tai Chi Qigong on: Cancer-related pain (Hedges’ g = −0.38, 95% CI −0.89 to 0.12, *p* = 0.136).
Wu 2019 [22]	Systematic review and Bayesian network meta-analysis	182 RCTs	18,491 cancer patients (45.05% studies on women with breast cancer, 25.27% studies on patients with multiple forms of cancer)	Qigong (style n.r.)	Placebo, usual care control, no intervention, waitlist control, or other non-pharmacological intervention	Cancer-related fatigue	Significant effect of Qigong on: Fatigue: lower level of fatigue compared to the control group (MD [95% CI], −2.03 [−3.36, −0.68])
Kuo 2021 [23]	Systematic review and meta-analysis	10 RCTs	811 cancer patients	Baduanjin Qigong	Routine care; active exercise	Cancer-related fatigue, sleep quality, QoL	Significant effect of Qigong on: Cancer-related fatigue: significantly less in the Baduanjin Qigong group compared with the control group (odds ratio = 0.27; 95% CI [0.17, 0.42]; test for overall effect: Z = 5.81, *p* < 0.00001; Heterogeneity I^2^ = 0%); QoL: Baduanjin Qigong exercise has a positive effect on breast cancer patients’ QoL: From EORTC-C30: MD = 13.13 (95% CI [1.87, 24.40]; Z = 2.29, *p* = 0.02; Heterogeneity I^2^ = 92%); From FACT-B: MD = 11.04 (95% CI [9.56, 12.53] Z = 14.57, *p* < 0.00001; Heterogeneity I^2^= 88%); Sleep Quality: Baduanjin Qigong exercise has a positive effect on breast cancer patients’ sleep quality: MD = −2.89 (95% CI [−3.48, −2.30] Z = 9.55, *p* < 0.001; Heterogeneity I^2^ = 0%).
Meng 2021 [24]	Systematic review and meta-analysis	14 RCTs; 3 CCTs	1236 breast cancer women	Chan-Chuang Qigong, Guolin New Qigong, Tai Chi Qigong, Kuala Lumpur Qigong, Sporting Qigong, Baduanjin	Any type of control	QoL, fatigue, sleep disturbance, cancer-related emotional disturbance	Significant effect of Qigong on: QoL: positive effect of Qigong compared to control procedures (n = 950, SMD = 0.65, 95% CI 0.23–1.08, *p* = 0.002); Depression: (n = 540, SMD = −0.32, 95% CI −0.59 to −0.04, *p* = 0.02, I^2^ = 59%); Anxiety-related serum protein (1 study): positive rate was significantly lower in the Qigong group than in the control group (*p* < 0.01); Immune responses (2 studies): significant reduction in IL-2, IFN-γ, IL-6, IL-1β, and TNF-α levels in Qigong group. No effect of Qigong on: Sleep disturbance: (n = 298, SMD = −0.11, 95% CI −0.74 to −0.52, *p* = 0.73, I^2^ = 86%); Cancer-related fatigue: (n = 206; SMD = −0.32, 95 % CI −0.71 to 0.07, *p* = 0.11, I^2^ = 73%); Anxiety: (n = 439, SMD = −0.71, 95% CI −1.32 to −0.10, *p* = 0.02, I^2^ = 89%).
Zeng 2014 [25]	Systematic review and meta-analysis	13 RCTs	592 cancer patients	Guolin Qigong, Medical Qigong	Any type of control	Body fat mass (BFM) and body mass index (BMI); anxiety and depression, biomarkers (cortisol levels, C-reactive protein (CRP)), and QOL	Significant effect of Qigong/Tai Chi on: Cancer-specific QOL (Z = 4.00 (*p* < 0.0001), I^2^ = 95%); Fatigue (Z = 2.09, *p* = 0.04, I^2^ = 90%); Immune function; Cortisol level: (Z = 1.97 (*p* = 0.05), I^2^ = 0%).
Cheung 2021 [26]	Systematic review and meta-analysis	11 RCTs	907 cancer patients	Guolin Qigong, Chan-Chuang Qigong, Xianggong, Baduanjin, General Qigong (style n.r.)	Placebo or usual care	Sleep quality and fatigue/depressive symptoms	Significant effect of Qigong on: Improving post-intervention: Sleep quality: (SMD = −1.28, 95% CI: −2.01, −0.55; *p* = n.r.; I^2^ = 95%); Fatigue: (SMD = −0.89, 95% CI: −1.59, −0.19; *p* = n.r.; I^2^ = 94%) in cancer patients post-intervention. Qigong’s effect on sleep was significantly mediated by its effect on fatigue (b = 1.27, SE = 0.24, *p* = 0.002), but not depressive symptoms (b = 0.53, SE = 0.26, *p* = 0.106). No effect of Qigong: Beneficial effects on sleep and fatigue became non-significant after 3 months. No effect of Qigong post-intervention on: Depressive symptoms: (SMD = −0.69, 95% CI: −1.81, 0.42; *p* = n.r.; I^2^ = 95%).
Lin 2019 [27]	Systematic review and meta-analysis	34 RCTs	3010 cancer patients (92% female)	Qigong (style n.r.)	No intervention	HRQoL	Significant improvement over usual care on: HRQOL - CH (MD = 6.03 [0.15–11.92]); - Qigong + MM (MD = 12.66 [8.75–16.57])
Ye 2022 [28]	Systematic review and meta-analysis	7 RCTs	450 postoperative breast cancer patients	Baduanjin Qigong	Any type of control	QoL, anxiety, depression	Significant effect of Qigong on: General QoL (FACT-B): (WMD = 5.70, 95% CI [3.11–8.29], Z = 4.32, *p* < 0.0001, I^2^ = 35%); Physical well-being (FACT-B): (WMD = 1.83, 95% CI [1.13, 2.53], Z = 5.15, *p* < 0.00001, I^2^ = 0%); Functional well-being (FACT-B): (WMD = 1.58, 95% CI [0.77–2.39], Z = 3.83 *p* = 0.0001, I^2^ = 0%); Role-physical QoL (SF-36): (WMD = 11.49, 95% CI [8.86, 14.13], Z = 8.55, *p* < 0.00001, I^2^ = 0%); Vitality (fatigue) (SF-36): (WMD = 8.58, 95% CI [5.60–11.56], Z = 5.65, *p* < 0.00001, I^2^ = 0%); Anxiety: (WMD = −8.02, 95% CI [−9.27- −6.78], Z = 12.62, *p* < 0.00001, I^2^ = 10%); Depression: (WMD = −4.45, 95%CI [−5.62–−3.28], Z = 7.45, *p* < 0.00001, I^2^ = 32%). No significant difference in: Social and emotional well-being (FACT-B); Physical functioning (SF-36); Bodily pain (SF-36); Social functioning (SF-36); General health (SF-36); Mental health (SF-36).
Liu 2022 [29]	Systematic review and network meta-analysis	12 RCTs	818 female breast cancer patients	Internal Qigong (style n.r.)	Routine nursing or treatments (e.g., placebo, usual care, no intervention, waitlist control, supportive therapy or other nonpharmacologic intervention)	Chemotherapy-related cognitive impairment (CRCI)	Significant effect of Qigong on: CRCI: improvement in the objective outcome of CRCI compared to psychotherapy (SMD = 1.27; 95% CI, 0.13–2.41) and to music therapy (SMD = 1.69; 95% CI, 0.10–3.28). No significant effect of Qigong compared with the non-pharmacological and control interventions on: CRCI: subjective outcomes.
Wang 2021 [30]	Systematic review and meta-analysis	16 RCTs	1313 patients (339 cancer patients)	Baduanjin, Six Healing Sounds Qigong, Wu Xing Ping Heng Gong, Shaolin Qigong Exercises, Self-improving exercise (style n.r.), Wuqinxi, Yijinjing	Any type of control	Fatigue	Significant effect of Qigong on: Cancer-related fatigue (total, physical, and mental): SMD−0.75 (−1.37 to−0.13) *p* = 0.02 I^2^ = 86%.
Zeng 2019 [31]	Systematic review and meta-analysis	12 RCTs	915 cancer patients	Qigong (style n.r.), Medical Qigong (style n.r.)	Usual care, support groups, waitlist control, or sham Qigong control	Treatment effects	Significant effect of Qigong on: Fatigue: (MD = 2.05, 95% CI [0.63, 3.47]; Z = 2.83 *p* = 0.005, I^2^ = 96%); Sleep difficulties (MD = 344.17, 95% CI [316.95, 371.39]; Z = 24.78, *p* = 0.00001, I^2^ 0%). No effect of Qigong on: Stress (MD = −8.56, 95% CI [−17.56, 0.44]; Z = 1.86, *p* = 0.06, I^2^ = 74%); Anxiety: (MD = −1.26, 95% CI [−3.73, 1.20]; Z = 1.00, *p* = 0.32, I^2^ = 43%); Depression: (MD = −2.58, 95% CI [−7.33, 2.17]; Z = 1.06, *p* = 0.29, I^2^ = 88%).

Notes: n.r. = not reported.

**Table 2:** Level of satisfaction of the 16 criteria required by AMSTAR2 quality assessment.

Number of Quality Assessment Criteria Satisfied by the Studies	Studies That Satisfy the Criteria
14/16	Klein et al., 2013 [13] Chan et al., 2012 [18] Meng et al., 2021 [24] Lin et al., 2019 [27]
13/16	Lee et al., 2007 [15] Farahani et al., 2022 [19] Wayne et., 2018 [21] Cheung et al., 2021 [26] Ye et al., 2022 [28] Wang et al., 2021 [30]
12/16	Henshall et al., 2019 [14] Van Vu et al., 2017 [17] Kuo et al., 2021 [23] Zeng et al., 2014 [25] Liu et al., 2022 [29]
11/16	Tao et al., 2016 [20]
10/16	Matthews et al., 2018 [16]
9/16	Wu et al., 2019 [22]
8/16	Zeng Y et al., 2019 [31]

**Table 3:** Studies’ quality assessment according to the 16 criteria defined by AMSTAR2.

Systematic Review/Meta-Analysis	Q1	Q2	Q3	Q4	Q5	Q6	Q7	Q8	Q9	Q10	Q11	Q12	Q13	Q14	Q15	Q16
Klein PJ et al. 2016 [13]	Y	N	Y	PY	Y	Y	Y	PY	Y	N	NA	NA	Y	Y	NA	Y
Henshall CL et al. 2019 [14]	Y	N	Y	PY	N	Y	N	PY	Y	N	Y	Y	Y	Y	Y	Y
Lee MS et al. 2007 [15]	Y	PY	N	Y	Y	Y	N	Y	Y	N	NA	NA	Y	Y	NA	Y
Matthews EE et al. 2018 [16]	Y	N	N	PY	Y	Y	N	Y	Y	N	NA	NA	Y	N	NA	N
Van Vu D et al. 2017 [17]	Y	PY	N	PY	Y	Y	N	PY	Y	N	NA	NA	Y	Y	NA	N
Chan CL et al. 2012 [18]	Y	Y	Y	PY	N	Y	PY	Y	PY	N	NA	NA	Y	Y	NA	Y
Farahani MA 2022 [19]	Y	PY	N	PY	Y	Y	N	PY	Y	N	NA	NA	Y	Y	NA	Y
Tao WW et al. 2016 [20]	Y	N	N	PY	Y	Y	N	PY	Y	N	Y	Y	Y	Y	N	Y
Wayne PM et al. 2018 [21]	Y	N	Y	PY	Y	Y	N	PY	Y	N	Y	Y	Y	Y	Y	Y
Wu C et al. 2019 [22]	Y	N	Y	PY	N	Y	N	N	Y	N	Y	Y	Y	N	N	Y
Kuo CC et al. 2021 [23]	Y	N	N	PY	Y	Y	N	PY	Y	N	Y	Y	Y	Y	Y	Y
Meng T et al. 2021 [24]	Y	N	Y	PY	Y	Y	N	Y	Y	Y	Y	Y	Y	Y	Y	Y
Zeng Y et al. 2014 [25]	Y	N	Y	PY	Y	N	N	PY	Y	N	Y	Y	Y	Y	Y	Y
Cheung DST et al. 2021 [26]	Y	N	Y	PY	Y	Y	N	Y	Y	N	Y	Y	Y	Y	Y	Y
Lin WF et al. 2019 [27]	Y	Y	Y	PY	Y	Y	N	Y	Y	N	Y	Y	Y	Y	Y	Y
Ye XX et al. 2022 [28]	Y	PY	N	PY	Y	Y	N	PY	Y	N	Y	Y	Y	Y	Y	Y
Liu Y et al. 2022 [29]	Y	PY	N	PY	Y	Y	N	PY	Y	N	Y	Y	Y	Y	Y	N
Wang R et al. 2021 [30]	Y	Y	N	PY	Y	Y	N	Y	Y	N	Y	Y	Y	Y	Y	Y
Zeng Y et al. 2019 [31]	Y	N	N	N	N	N	N	PY	Y	N	Y	Y	Y	Y	N	Y

The table describes the compliance of the studies with the AMSTAR2 16 criteria, evaluated through 16 questions reported below. Q1—Did the research questions and inclusion criteria for the review include the components of PICO? Q2—Did the report of the review contain an explicit statement that the review methods were established prior to the conduct of the review and did the report justify any significant deviations from the protocol? Q3—Did the review authors explain their selection of the study designs for inclusion in the review? Q4—Did the review authors use a comprehensive literature search strategy? Q5—Did the review authors perform study selection in duplicate? Q6—Did the review authors perform data extraction in duplicate? Q7—Did the review authors provide a list of excluded studies and justify the exclusions? Q8—Did the review authors describe the included studies in adequate detail? Q9—Did the review authors use a satisfactory technique for assessing the risk of bias (RoB) in individual studies that were included in the review? Q10—Did the review authors report on the sources of funding for the studies included in the review? Q11—If meta-analysis was performed, did the review authors use appropriate methods for statistical combination of results? Q12—If meta-analysis was performed, did the review authors assess the potential impact of RoB in individual studies on the results of the meta-analysis or other evidence synthesis? Q13—Did the review authors account for RoB in primary studies when interpreting/discussing the results of the review? Q14—Did the review authors provide a satisfactory explanation for, and discussion of, any heterogeneity observed in the results of the review? Q15—If they performed quantitative synthesis did the review authors carry out an adequate investigation of publication bias (small study bias) and discuss its likely impact on the results of the review? Q16—Did the review authors report any potential sources of conflict of interest, including any funding they received for conducting the review? Responses to the criteria questions are Y = Yes, that is, complete compliance with the criterion as defined by AMSTAR2; PY = Partially Yes, that is, partial compliance with the criterion as defined by AMSTAR2; N = No, that is, no compliance with the criterion as defined by AMSTAR2; NA = Not Applicable.

**Table 4:** Summary of evidence about psychological and clinical outcomes.

Outcome	Evidence Supporting Qi Gong Integration
Quality of life	YES
Sleep quality	YES *
Cancer-related fatigue	YES
Depression	Uncertain
Anxiety	Uncertain
Stress/distress	NO
Cognitive impairment	YES **
Cancer-related symptoms: dyspnea	NO
Cancer-related symptoms: pain	NO
Cancer-related symptoms: nausea and vomiting	YES *
Cancer-related symptoms: appetite, bowel function	YES *

The table summarizes the evidence in favor or against the effect of Qigong intervention on psychological and clinical outcomes. YES = most of the evidence supports the effect of Qigong; Uncertain = the evidence is contradictory on the effect of Qi Gong; No = most of the evidence does not support the effect of Qigong. * Dosage and/or timing dependent; ** favoring objective measures.

**Table 5:** Summary of evidence about biological outcomes.

Biological Marker	Meaning	Evidence Supporting Qigong Integration
Cortisol level	Hypothalamic–pituitary–adrenal activity	NO
IL-2	Inflammation response	Uncertain
IL-6	Inflammation response	Uncertain
TNF-a	Inflammation response	Uncertain
CRP	Inflammation response	YES *
IFN-y	Inflammation response	NO
WBC	Immune response	YES
RBC, Hb	Erythropoiesis	YES
α-Naphthyl butyrate esterase (ANAE)	Immune response	YES

The table summarizes the evidence in favor or against the effect of Qigong intervention on biological outcomes. YES = most of the evidence supports the effect of Qigong; Uncertain = the evidence is contradictory on the effect of Qi Gong; No = most of the evidence does not support the effect of Qigong. * In Qigong programs longer than 8 weeks.
